# Long-Term Effects of Right Ventricular Pressure and Volume Overload: A Comparative Analysis in an Ovine Model

**DOI:** 10.3390/jcm15176836

**Published:** 2026-09-03

**Authors:** Hamida Al Hussein, Hussam Al Hussein, Simina-Elena Ghiragosian-Rusu, Simona Gurzu, Rita Szodorai, Carmen Sircuta, Marius Stefan Marusteri, Bogdan Cordos, David Emanuel Anitei, Andrei Atudorei, Sergiu Crisan, Dan Simionescu, Klara Brinzaniuc

**Affiliations:** 1 Doctoral School, George Emil Palade University of Medicine, Pharmacy, Science and Technology of Targu Mures, 38 Gheorghe Marinescu Street, 540142 Targu Mures, Romania; hamida.alhussein@yahoo.com; 2 Department of Anesthesiology and Critical Care, Clinical County Hospital Mures, 1 Gheorghe Marinescu Street, 540103 Targu Mures, Romania; 3 Department of Anatomy and Embryology, George Emil Palade University of Medicine, Pharmacy, Science and Technology of Targu Mures, 38 Gheorghe Marinescu Street, 540142 Targu Mures, Romania; klara_branzaniuc@yahoo.com; 4 Regenerative Medicine Laboratory, Center for Advanced Medical and Pharmaceutical Research, George Emil Palade University of Medicine, Pharmacy, Science and Technology of Targu Mures, 38 Gheorghe Marinescu Street, 540139 Targu Mures, Romania; exoticvet_umf@yahoo.com (B.C.); dsimion@clemson.edu (D.S.); 5 Department of Cardiovascular Surgery, Emergency Institute for Cardiovascular Diseases and Transplantation, 50 Gheorghe Marinescu Street, 540136 Targu Mures, Romania; anitei_emanuel@yahoo.com; 6 Department of Pediatrics III, George Emil Palade University of Medicine, Pharmacy, Science and Technology of Targu Mures, 38 Gheorghe Marinescu Street, 540142 Targu Mures, Romania; simina_r88@yahoo.com; 7 Department of Pediatric Cardiology, Emergency Institute for Cardiovascular Diseases and Transplantation, 50 Gheorghe Marinescu Street, 540136 Targu Mures, Romania; 8 Department of Pathology, George Emil Palade University of Medicine, Pharmacy, Science and Technology of Targu Mures, 38 Gheorghe Marinescu Street, 540142 Targu Mures, Romania; simona.gurzu@umfst.ro (S.G.); ritaszodorai@gmail.com (R.S.); 9 Research Center of Oncopathology and Translational Research (CCOMT), George Emil Palade University of Medicine, Pharmacy, Science and Technology of Targu Mures, 38 Gheorghe Marinescu Street, 540139 Targu Mures, Romania; 10 Department of Anesthesiology and Critical Care, Emergency Institute for Cardiovascular Diseases and Transplantation, 50 Gheorghe Marinescu Street, 540136 Targu Mures, Romania; carmensircuta@gmail.com; 11 Department of Medical Informatics and Biostatistics, George Emil Palade University of Medicine, Pharmacy, Science and Technology of Targu Mures, 38 Gheorghe Marinescu Street, 540142 Targu Mures, Romania; marius.marusteri@umfst.ro; 12 Center of Experimental and Imaging Studies, George Emil Palade University of Medicine, Pharmacy, Science and Technology of Targu Mures, 38 Gheorghe Marinescu Street, 540139 Targu Mures, Romania; sergiucrisan5@gmail.com; 13 Department of Anesthesiology and Critical Care, Ovidius Clinical Hospital, DN2A km 202 + 880, Ovidiu, 905900 Constanta, Romania; andreileu8@yahoo.com; 14 Biocompatibility and Tissue Regeneration Laboratory, Department of Bioengineering, Clemson University, Sikes Hall, Clemson, SC 29634, USA

**Keywords:** right ventricular failure, pressure overload, volume overload, hemodynamic assessment, ovine model

## Abstract

**Background/Objectives**: Pressure overload (PO) and volume overload (VO) represent important mechanisms underlying right ventricular (RV) failure; however, long-term comparative data regarding their distinct pathophysiological consequences remain limited. This study aimed to compare the long-term effects of PO versus VO in a large animal model. **Methods**: Juvenile sheep were randomly assigned to either a PO group (*n* = 7) or a VO group (*n* = 8). PO was induced by pulmonary artery banding, whereas VO was induced by pulmonary leaflet perforation (*n* = 4) or transannular patch (TAP) reconstruction (*n* = 4). Both VO procedures resulted in moderate-to-severe pulmonary regurgitation with comparable acute hemodynamic profiles and were therefore analyzed as a single VO group for the long-term assessment. Six animals from each group completed the follow-up period. Animals were evaluated preoperatively and at one-year follow-up using invasive hemodynamic monitoring, blood gas analysis, and echocardiography, followed by postmortem histological analysis of the myocardium. **Results**: Both models resulted in significant reductions in stroke volume index, cardiac index, RV stroke work index, ScvO_2_ and tricuspid annular plane systolic excursion (TAPSE). Similarly, the TAPSE/RV systolic pressure (RVSP) ratio decreased in both groups but was significantly lower in the PO group than in the VO group (*p* = 0.0022). Moreover, PO showed more pronounced RV diastolic dysfunction, characterized by a significant increase in right atrial pressure from baseline (*p* = 0.0313) and higher RV diastolic pressure compared to VO (*p* = 0.0065). In contrast, VO showed a greater impact on left ventricular (LV) geometry and diastolic filling, evidenced by reduced transmitral filling (*p* = 0.0313) and a lower indexed LV internal diameter compared to PO (*p* = 0.0411). Histological analysis revealed myocardial hypertrophy and fibrosis in both groups, as well as clusters of multinucleated cardiomyocyte-like cells showing preferential ventricular distribution according to overload physiology. **Conclusions**: Chronic PO and VO induced distinct phenotypes of RV dysfunction with differential ventricular adaptation and remodelling patterns.

## 1. Introduction

The function and clinical importance of the right ventricle (RV) have historically received limited attention, despite being first described by William Harvey in 1628 [1]. However, accumulating evidence over the past decades has highlighted its prognostic significance across a broad spectrum of cardiovascular diseases, including left ventricular (LV) failure, pulmonary hypertension (PH), cardiomyopathies, and congenital heart disease (CHD) [2,3].

Despite extensive research, findings derived from left ventricular failure (LVF) cannot be directly extrapolated to right ventricular failure (RVF). In contrast to the LV, the RV operates within a low-pressure circulation, requiring significantly less stroke work to maintain forward flow. Structurally, it is a thin-walled, highly compliant chamber capable of accommodating substantial variations in preload with minimal changes in filling pressures [2,4]. These unique anatomical and physiological characteristics result in distinct adaptive and maladaptive responses to hemodynamic stress.

Among the mechanisms leading to RVF, chronic pressure overload (PO) and volume overload (VO) represent two clinically relevant yet pathophysiologically distinct forms of hemodynamic stress. While both conditions ultimately promote RV remodeling and progressive dysfunction, ventricular adaptation differs substantially between the two. Understanding these differences is clinically relevant, as the mechanisms and temporal progression from adaptive remodeling to RVF may differ depending on the type of hemodynamic load [5], with potential implications for patient surveillance, recognition of disease progression, and the optimal timing of therapeutic intervention [6]. However, long-term comparative data remain limited.

In this context, large animal models play an important role in preclinical research. Among these, sheep have been widely utilized across a broad spectrum of experimental studies, encompassing fields as diverse as regenerative medicine, translational cardiovascular research, and experimental heart failure models [7,8,9,10,11,12]. Their docile behaviour, ease of handling, and important anatomical, hemodynamic, and metabolic similarities to humans [13,14] make them a valuable translational platform for evaluating innovative therapeutic strategies [15,16].

The aim of this study was therefore to characterize the long-term hemodynamic, echocardiographic and histopathological consequences of chronic RV pressure and volume overload in an ovine model and to evaluate the distinct pathophysiological mechanisms involved in the progression toward RVF.

## 2. Materials and Methods

### 2.1. Animals and Experimental Design

Ethical approval for this study was obtained from the Scientific Research Ethics Committee of the George Emil Palade University of Medicine, Pharmacy, Science, and Technology of Targu Mures, with approval no. 1451/dated 22 July 2021. The study was further approved by the Mures Sanitary Veterinary and Food Safety Department (approval no. 54/dated 31 August 2022). All procedures and perioperative care were performed in accordance with the Guidelines for the Care and Use of Laboratory Animals and Directive 2010/63/EU of the European Parliament on the protection of animals used for scientific purposes. Experimental procedures were conducted in accordance with the 3R principles (replacement, reduction, and refinement), with efforts made to minimize animal use and optimize perioperative analgesia to ensure animal welfare.

The study was conducted on juvenile male and female sheep (Romanian Tsigai breed), aged 12–18 weeks and weighing 18–42 kg, randomly assigned to either a PO group (*n* = 7; 5 females and 2 males) or a VO group (*n* = 8; 5 females and 3 males). Details regarding anesthetic management, catheter insertion techniques, surgical protocols, and acute hemodynamic findings have been reported previously [17]. Briefly, all procedures were performed off-pump through a left thoracotomy under volatile-based general anesthesia. PO was induced by pulmonary artery banding (PAB) using a Gore-Tex band. The pulmonary artery was progressively narrowed under continuous monitoring of invasive systemic blood pressure and RV pressure through the Swan–Ganz catheter. We targeted an RV pressure of approximately two-thirds of systemic blood pressure, with further adjustments according to the hemodynamic tolerance of each animal. Postoperatively, the peak pressure gradient across the band was assessed echocardiographically, with a mean value of 44.3 mmHg obtained across the group. VO was induced by creating pulmonary regurgitation (PR), either through pulmonary leaflet perforation (*n* = 4) or pulmonary annulotomy with transannular patch (TAP) reconstruction (*n* = 4). Pulmonary leaflet perforation was performed using a 6-mm aortic punch, whereas TAP reconstruction was performed using an approximately 2-cm-wide heterologous pericardial patch. Moderate-to-severe PR was achieved with both procedures. PR severity was quantified echocardiographically using the PR jet width/right ventricular outflow tract (RVOT) diameter ratio, which was 0.58 in the TAP group and 0.51 in the leaflet perforation group. Given the comparable PR severity and acute hemodynamic profiles observed following the two procedures [17], both subgroups were subsequently analyzed as a single VO group.

For the comparative analysis presented in this study, all parameters, including demographic data, were recorded at two predefined timepoints: preoperatively and at one-year follow-up prior to euthanasia. Six animals in each group completed the one-year follow-up (4 females and 2 males in each group).

To ensure comparability between timepoints, all measurements were performed under standardized physiological conditions. Hemodynamic measurements and blood gas analyses were performed under general anesthesia, while transthoracic echocardiography was performed in conscious sheep positioned in right lateral recumbency.

### 2.2. Hemodynamic and Functional Assessment

Invasive hemodynamic measurements were obtained using an arterial catheter placed in the left auricular or tibial artery and a Swan–Ganz catheter inserted through the left external jugular vein. Recorded parameters included systolic, diastolic, and mean arterial pressures (SBP, DBP, and MAP); right ventricular systolic (RVSP), diastolic (RVDP), and mean (mRVP) pressures; pulmonary artery systolic (sPAP), diastolic (dPAP), and mean (mPAP) pressures; right atrial pressure (RAP); pulmonary capillary wedge pressure (PCWP); cardiac index (CI); stroke volume index (SVI); systemic and pulmonary vascular resistance indices (SVRI and PVRI); right and left ventricular stroke work indices (RVSWI and LVSWI); and right and left cardiac work indices (RCWI and LCWI).

Blood gas analyses were performed to assess oxygenation, acid–base balance, and metabolic status. Measured parameters included pH, partial pressures of oxygen and carbon dioxide (pO_2_ and pCO_2_), arterial oxygen saturation (SaO_2_), bicarbonate (HCO_3_^−^), base excess (BE), lactate, hemoglobin (Hb), hematocrit (Hct), and central venous oxygen saturation (ScvO_2_). Arterial blood samples were collected from either tibial or auricular arterial lines, whereas ScvO_2_ was measured via the distal lumen of the central venous catheter.

Transthoracic echocardiography (TTE) included assessment of right and left ventricular functional parameters. Tricuspid annular plane systolic excursion (TAPSE) and mitral annular plane systolic excursion (MAPSE) were measured using M-mode imaging in the apical four-chamber view. Diastolic transtricuspid and transmitral early-to-late flow velocity ratios (E/A ratio) were assessed using pulsed-wave Doppler in the apical four-chamber view. Left ventricular internal diameter (LVIDd) and interventricular septum (IVSd) thickness at end-diastole were measured using M-mode imaging obtained from the parasternal long-axis (PLAX) view. Right ventricular internal diameter at end-diastole (RVIDd) was measured in the apical four-chamber view. LVIDd and RVIDd values were indexed to body surface area (BSA) to account for somatic growth during the one-year follow-up period and differences in body size. Left ventricular ejection fraction (LVEF) and fractional shortening (LVFS) were calculated using the Teichholz formula. Abnormal septal motion was assessed qualitatively by a single experienced cardiologist. The TAPSE/RVSP ratio was used as a surrogate measure of RV–pulmonary arterial (RV-PA) coupling.

### 2.3. Tissue Collection and Morphological Assessment

At the completion of the one-year follow-up period, animals were euthanized under deep general anesthesia with intravenous pentobarbital (140 mg/kg), in accordance with institutional protocols and applicable European regulations governing the use of animals for scientific research. Following euthanasia, hearts were excised and weighed to obtain heart weight (HW), right ventricular weight (RVW), and combined left ventricular plus interventricular septal weight (LV + IVS). To account for somatic growth during the follow-up period, ventricular weights were also indexed to BSA (RVW/BSA and LV + IVS/BSA). In addition, RVW/HW, LV + IVS/HW, and RVW/LV + IVS (Fulton index) were calculated to assess ventricular structural remodeling.

Tissue samples for histological examination were collected from the same predefined mid-ventricular regions of the RV free wall, IVS, and LV free wall in all animals. Samples were fixed in formalin, embedded in paraffin, sectioned at 5 µm, and processed for microscopy analysis. Hematoxylin and eosin stain was performed for overall morphology assessment of cardiomyocytes and the density of inflammatory cells (cells/mm^2^), while Masson’s trichrome and Pas-Alcian stains were used to estimate the fibrotic areas and hyalinization. Immunohistochemical stains included desmin (clone D33 ready to use, from Agilent, Santa Clara, CA, USA), for evaluation of the structural integrity of cardiomyocytes and the proliferation marker Ki-67 (clone MIB-1 ready to use, from Agilent, Santa Clara, CA, USA).

Quantification of the number of desmin-positive clusters of multinucleated cardiomyocyte-like cells was firstly done with the light microscope with an attached digital camera, by two pathologists (S.G., R.S.). Quantitative analysis across 20 randomly selected non-overlapping high-power fields (200×) was performed. The manual scoring was completed by digital image analysis using a high-resolution whole-slide scanner (PANNORAMIC 250 Flash III; 3DHISTECH Ltd., Budapest, Hungary) with dedicated quantitative pathology software (QuantCenter version 3.0; 3DHISTECH Ltd., Budapest, Hungary) [18].

### 2.4. Statistical Analysis

All data are presented as mean ± standard deviation (SD), with median values additionally reported. Statistical analysis was performed using GraphPad Prism version 10.4.0 (GraphPad Software, Boston, MA, USA). Given the small sample size, nonparametric statistical methods were used for all comparisons. The Wilcoxon signed-rank test was used for within-group comparison between baseline and one-year follow-up measurements, while the Mann–Whitney U test was used for between-group comparisons. Spearman’s rank correlation analysis was used to assess associations between structural, hemodynamic, and echocardiographic parameters within the PO group. Graphical representations were generated using IBM SPSS Statistics version 32.0 (IBM Corp., Armonk, NY, USA). Statistical significance was defined as *p* < 0.05.

## 3. Results

Demographic characteristics are summarized in Table 1. Both groups showed increased body weight, height, and BSA at one-year follow-up compared to baseline, with no significant differences observed between the groups.

### 3.1. Early and Late Mortality

One animal (female) in the PO group died within the first 12 postoperative hours, as previously described [17]. This animal had developed intraoperative atrial fibrillation, which did not require pharmacological treatment, as it spontaneously converted to sinus rhythm. Gross post-mortem and histopathological examination revealed no significant findings; therefore, an arrhythmic event was considered a possible cause of death, although continuous 24-h postoperative ECG monitoring was not available to confirm this hypothesis. Two additional animals (one female and one male) in the VO group died during the follow-up period, at 5 and 7 months postoperatively, after developing signs of cardiac decompensation, including reduced activity, inappetence, and respiratory distress. These animals were therefore excluded from the one-year comparative analysis.

### 3.2. Effects of Chronic PO and VO on Right Ventricular Systolic Function and RV-PA Coupling

Detailed hemodynamic and blood gas parameters are summarized in Table 2 and Table 3, respectively. One-year exposure to both loading conditions resulted in a significant increase in RVSP. However, this increase was greater in the PO group than in the VO group (51.7 ± 7.3 vs. 33.0 ± 3.3 mmHg, *p* = 0.0022). SVI was significantly lower at follow-up in both groups, decreasing from 58.4 ± 14.5 to 30.7 ± 4.2 mL/m^2^, (*p* = 0.0313) in the PO group and from 58.6 ± 10 to 31.3 ± 14.7 mL/m^2^ (*p* = 0.0313) in the VO group, without significant differences between the groups. Similar reductions in CI, RVSWI and RCWI were observed in both models. Likewise, ScvO_2_ declined significantly in both groups compared to baseline. These findings were further supported by the significant decline in TAPSE, which showed comparable changes in both groups, decreasing from 16.3 ± 1.1 to 8.8 ± 1.2 mm (*p* = 0.0313) in the PO group and from 14.9 ± 3.2 to 9.8 ± 1.3 mm (*p* = 0.0313) in the VO group. When accounting for afterload and RV-PA coupling, the TAPSE/RVSP ratio also declined in both groups at follow-up; however, PO showed a significantly lower ratio compared to VO (0.17 ± 0.02 vs. 0.30 ± 0.06, *p* = 0.0022) (Table 4).

### 3.3. Effects of Chronic PO and VO on Right Ventricular Diastolic Function

Detailed echocardiographic parameters are summarized in Table 4. Assessment of RV diastolic function at one-year follow-up revealed reduced transtricuspid E/A ratios in both groups (PO: 1.13 ± 0.15 vs. 0.88 ± 0.11, *p* = 0.0313; VO: 1.47 ± 0.27 vs.0.76 ± 0.12, *p* = 0.0313), consistent with an impaired relaxation pattern. However, in contrast to VO, chronic PO resulted in elevated filling pressures. RVDP more than doubled compared to baseline in the PO group (4.2 ± 2.8 vs. 9.3 ± 1.0 mmHg, *p* = 0.0313) and was significantly higher than in the VO group at follow-up (9.3 ± 1.0 vs. 4.7 ± 2.7 mmHg, *p* = 0.0065). Similarly, RAP increased approximately two-fold in the PO group (6.8 ± 1.6 vs. 13.3 ± 5.0 mmHg, *p* = 0.0313), whereas the VO group showed a 60% increase that did not reach statistical significance (*p* = 0.0625). No significant correlation was found between transtricuspid E/A ratio and RAP (Spearman’s ρ = −0.0857, *p* = 0.9194) or RVDP (Spearman’s ρ = 0.1518, *p* = 0.8028) in the PO group.

### 3.4. Left Ventricular Function and Ventricular Interdependence

**Figure 1:**
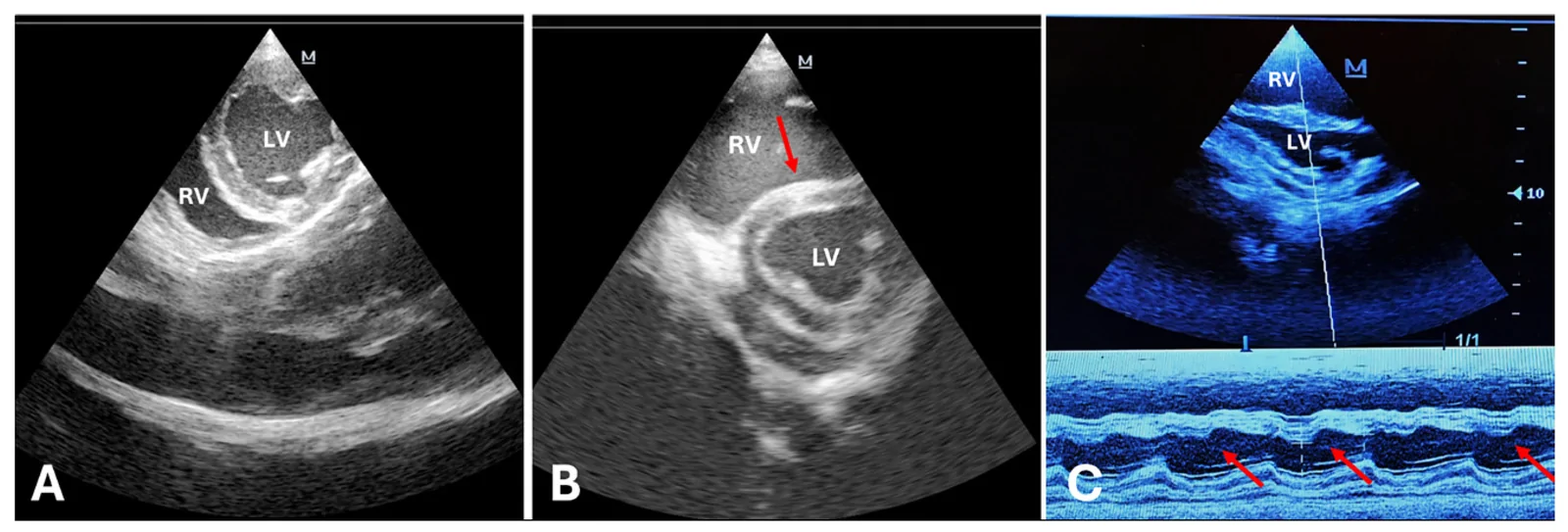
Transthoracic echocardiography showing: (**A**) Normal septal motion in an animal from the volume overload (VO) group at baseline (preoperative), visualized in parasternal short-axis (PSAX) view at the mid-papillary level. (**B**) The same animal at one-year follow-up showing right ventricular dilation and leftward septal shift at end-diastole (red arrow). (**C**) M-mode imaging obtained from the parasternal long-axis (PLAX) view in an animal from the pressure overload (PO) group at one-year follow-up, showing right ventricular dilation and abnormal septal motion with posterior septal bowing during diastole (red arrows); RV—right ventricle; LV—left ventricle.

Echocardiographic assessment revealed abnormal septal motion in all animals from both groups, characterized by leftward shift in the interventricular septum (IVS) (Figure 1). This was associated with reduced LVIDd index in both groups compared to baseline. However, LVIDd index was significantly lower in the VO group compared to the PO group (2.7 ± 0.9 vs. 3.8 ± 0.6 cm/m^2^, *p* = 0.0411). In addition, transmitral E/A ratio decreased exclusively in the VO group (1.35 ± 0.26 vs. 0.98 ± 0.1, *p* = 0.0313) and was significantly lower than in the PO group (0.98 ± 0.10 vs. 1.35 ± 0.26, *p* = 0.0152). The impact on LV function was further supported by reduced LVSWI observed in both groups. Furthermore, DBP and MAP decreased in both groups, although statistical significance was reached only in the VO group (Table 2). Conventional echocardiographic parameters of LV systolic function remained largely preserved, with only a 22.4% reduction in MAPSE observed in the VO group that did not reach statistical significance (*p* = 0.0625), while LVEF and LVFS remained unchanged in both groups.

### 3.5. Structural Remodeling Patterns Induced by Chronic PO and VO

**Figure 2:**
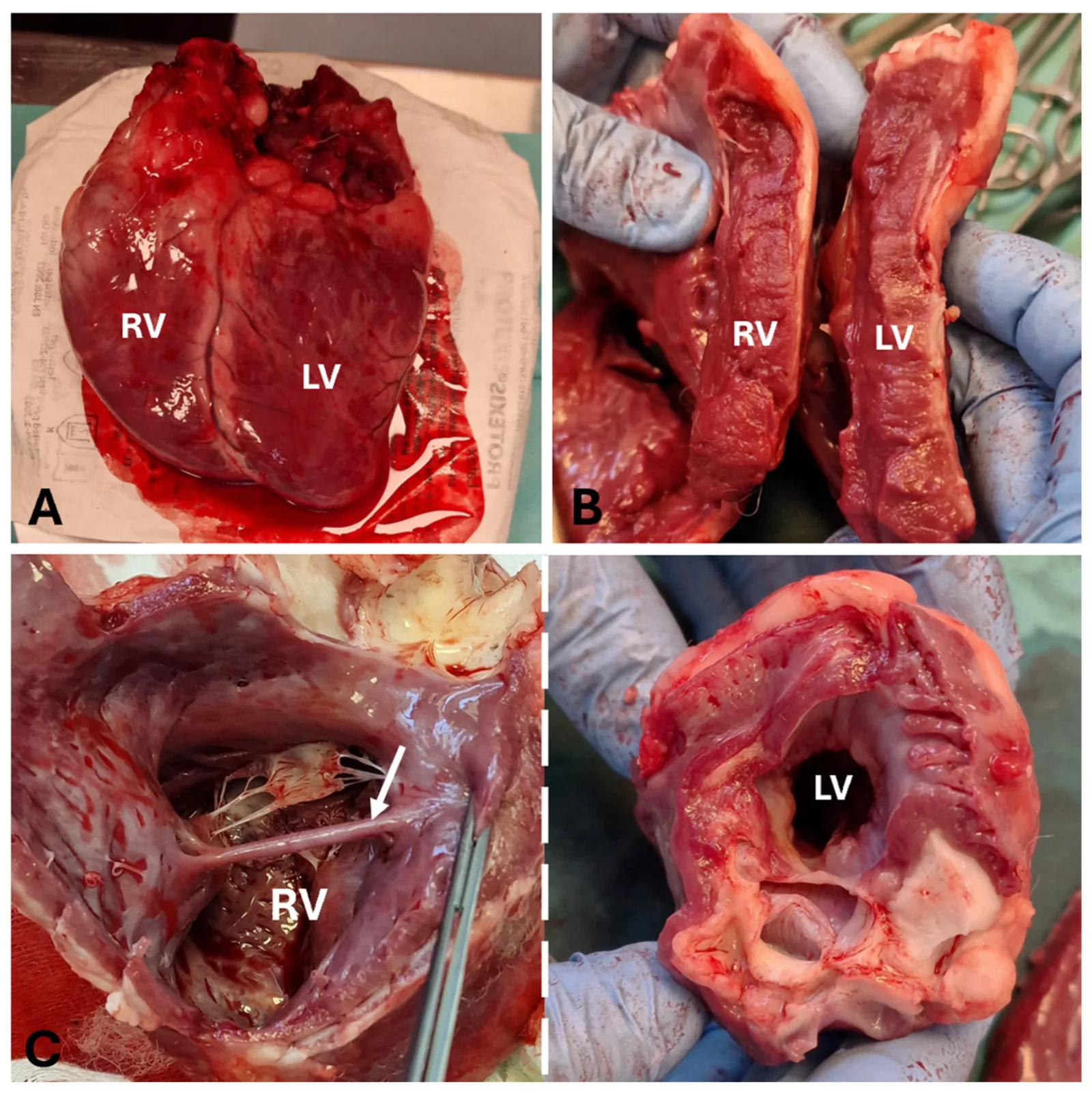
Macroscopic evaluation of the explanted hearts showing: (**A**) Enlarged right ventricle (RV) in an animal from the pressure overload (PO) group. (**B**) Hypertrophy of the RV inferolateral wall (left) compared with the left ventricular (LV) free wall (right). (**C**) Representative images from an animal in the volume overload (VO) group showing the moderator band (arrow) and an enlarged RV cavity in a section through the RV anterior wall (**left**), compared with the corresponding LV cavity of the same animal visualized in a section at the level of the mitral annulus (**right**).

**Figure 3:**
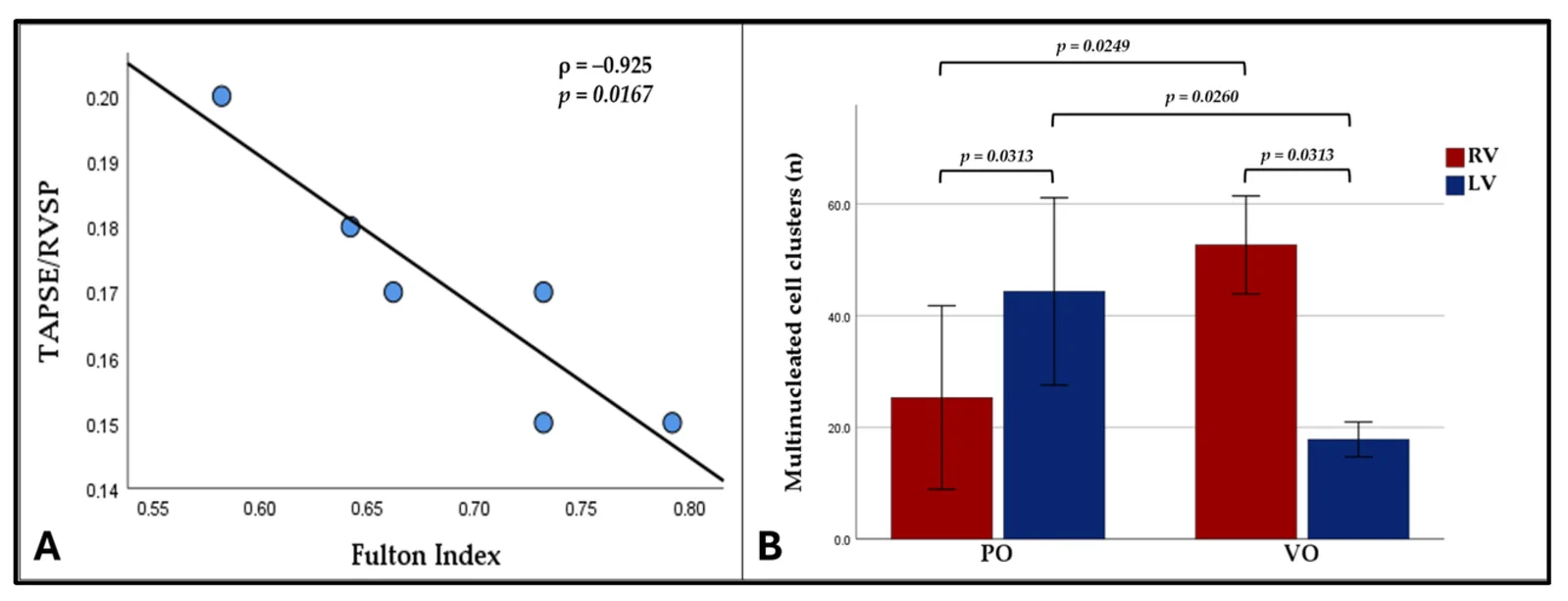
(**A**) Correlation between Fulton index and TAPSE/RVSP ratio in the pressure overload (PO) group at one-year follow-up. Spearman’s rank correlation analysis demonstrated a strong inverse relationship between the Fulton index and TAPSE/RVSP ratio. Blue circles represent individual animals, and the black line represents the linear regression line. (**B**) Distribution of multinucleated cell clusters in the right ventricle (RV) and left ventricle (LV) of animals exposed to chronic pressure overload (PO) and volume overload (VO). Data are presented as mean ± standard deviation. Within-group comparisons (RV vs. LV) were performed using the Wilcoxon signed-rank test, while between-group comparisons (PO RV vs. VO RV and PO LV vs. VO LV) were performed using the Mann–Whitney U test.

Chronic exposure to both loading conditions resulted in increased IVSd thickness (PO: 0.71 ± 0.14 vs. 1.14 ± 0.14 cm, *p* = 0.0313; VO: 0.86 ± 0.17 vs.1.14 ± 0.06 cm, *p* = 0.0313), without significant differences between the groups (Table 4). At one-year follow-up, RVIDd index was higher in the VO group than in the PO group (2.7 ± 0.7 vs. 2.2 ± 0.7 cm/m^2^), although the difference did not reach statistical significance. RVIDd was not determined at baseline, therefore precluding longitudinal comparison. Macroscopic structural characteristics at follow-up are summarized in Table 5. Gross examination of the explanted hearts revealed enlarged right ventricles in all animals (Figure 2A), with marked RV wall thickening in the PO group (Figure 2B), whereas the VO group predominantly showed RV dilatation (Figure 2C). While HW showed only an increasing trend in the PO group (*p* = 0.0649), RVW and the RVW/HW ratio were higher compared to the VO group (RVW: 93.2 ± 24.4 vs. 65.2 ± 13.0 g, *p* = 0.0260; RVW/HW: 0.40 ± 0.02 vs. 0.33 ± 0.04, *p* = 0.0200). Similarly, the Fulton index was approximately 40% higher in the PO group than in the VO group (0.69 ± 0.08 vs.0.50 ± 0.10, *p* = 0.0087). Moreover, a strong inverse correlation between the Fulton index and the TAPSE/RVSP ratio was identified within the PO group (Spearman’s ρ = −0.925, *p* = 0.0167) (Figure 3A). In contrast, the Fulton index did not significantly correlate with transtricuspid E/A ratio (Spearman’s ρ = −0.5508, *p* = 0.2972) or RVDP (Spearman’s ρ = 0.000, *p* > 0.9999) in the PO group.

**Figure 4:**
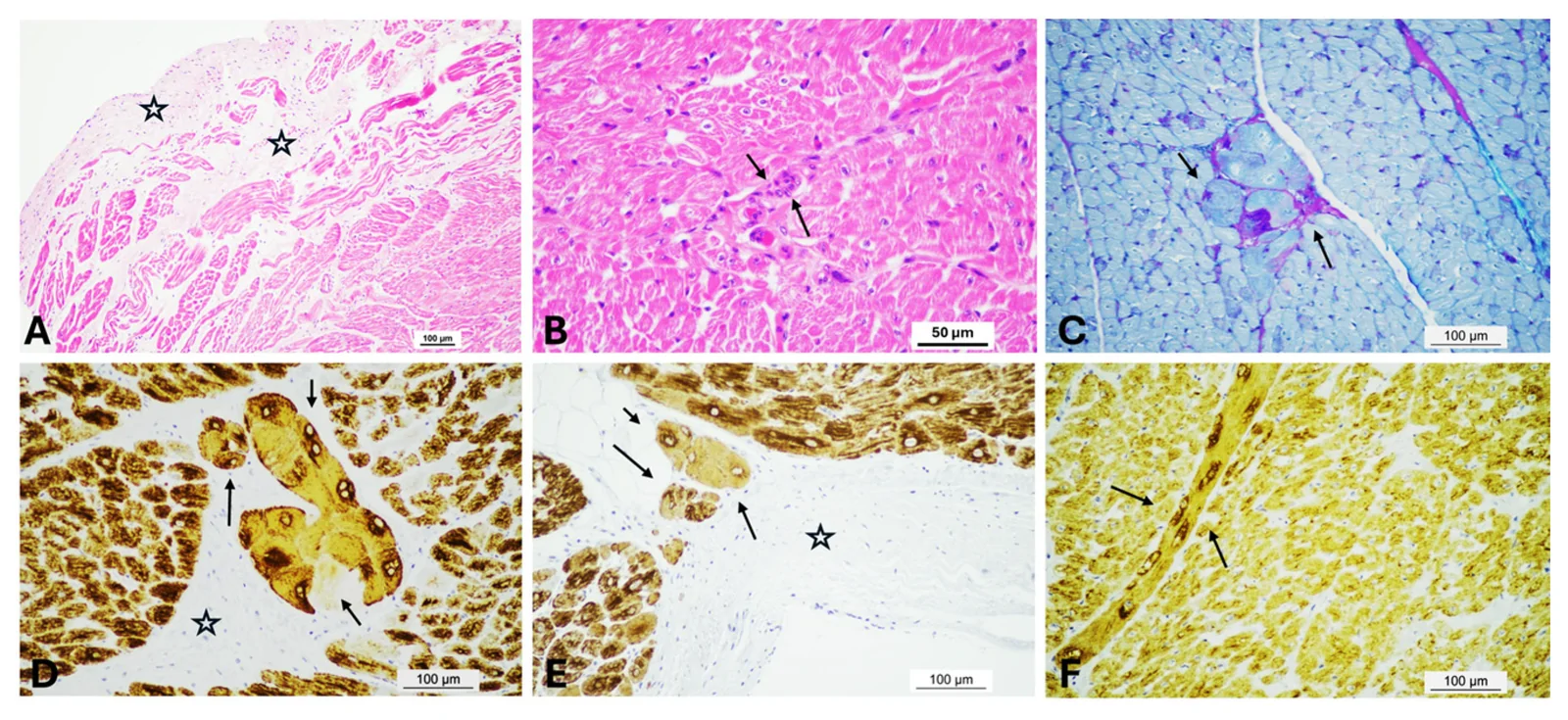
Representative histological sections from the myocardium of animals exposed to chronic pressure overload (PO) and volume overload (VO). (**A**) Subendocardial fibrosis (star). (**B**) Formation of multinucleated cell clusters between the myocardial fibers (arrows), without fibrosis. (**C**) PAS-Alcian staining of the multinucleated cells (arrows). (**D**,**E**) Fibrosis (star) and desmin-positive multinucleated cells (arrows). (**F**) Desmin-positive multinucleated cells between the myocardial fibers (arrows), without fibrosis. Sections were stained with hematoxylin and eosin (**A**,**B**), PAS–Alcian (**C**), and immunohistochemically for desmin (**D**–**F**); desmin-positive cells appear brown.

Histological assessment confirmed cardiomyocyte hypertrophy and interstitial fibrosis within the RV myocardium in both groups. In addition, clusters of desmin-positive/Ki67 negative large multinucleated cells, morphologically resembling cardiomyocytes, were identified in both ventricles (Figure 4). Quantitative analysis demonstrated distinct ventricular distribution patterns of the giant cells, depending on the underlying overload physiology. The PO group showed a higher frequency of these clusters in the LV compared to the RV (44.3 ± 16.8 vs. 25.3 ± 16.4, *p* = 0.0313), whereas the VO group showed the opposite pattern, with greater cluster frequency in the RV compared to the LV (52.7 ± 8.8 vs. 17.8 ± 3.1, *p* = 0.0313) (Figure 3B). The inflammatory cells, mainly lymphocytes, were only scattered between the muscle fibers without being connected with the giant multinucleated cells.

## 4. Discussion

### 4.1. Principal Findings

In this study, we described the long-term hemodynamic, echocardiographic and histopathological consequences of chronic RV PO and VO in an ovine model. To our knowledge, this is the longest follow-up reported in a preclinical large animal model comparing these two loading conditions. The principal findings were: (1) chronic exposure to both PO and VO resulted in significant impairment of RV systolic function and reduced TAPSE/RVSP ratio; (2) PO demonstrated RV diastolic dysfunction reflected by elevated filling pressures, whereas VO showed predominantly impaired RV relaxation without a significant increase in RVDP; (3) both experimental groups developed secondary LV diastolic dysfunction, but VO resulted in a more pronounced left ventricular involvement, characterized by significantly lower LVIDd index as well as impaired transmitral filling; (4) prolonged exposure to both loading conditions resulted in structural remodeling characterized by histological evidence of myocardial hypertrophy and fibrosis; and (5) large multinucleated cardiomyocyte-like cell clusters were identified in both models, showing preferential ventricular distribution according to the type of overload, suggesting the presence of a potentially novel adaptive or maladaptive remodeling mechanism.

### 4.2. Right Ventricular Systolic Dysfunction and RV-PA Uncoupling

One year following induction of overload, both pressure- and volume-overloaded ventricles demonstrated significant impairment of RV systolic performance, evidenced by decreased SVI and CI compared with baseline values, accompanied by reductions in RVSWI and RCWI. Consistent with our findings, several experimental studies have reported RV systolic dysfunction following chronic PO, characterized by reduction in stroke volume, cardiac output and RV ejection fraction [19,20]. Interestingly, these changes occurred despite increased RV contractility indices, likely reflecting failure of the compensatory increase in contractility to preserve forward flow [19,20]. In contrast, the effects of chronic VO on RV systolic function remain more controversial. Several investigators reported relatively preserved systolic function at rest despite evidence of impaired contractile reserve [21,22]. An important distinction, however, is the duration of follow-up, as most experimental studies evaluated animals after weeks or several months of overload, whereas our model allowed assessment after one year of continuous volume loading. Nevertheless, our findings also appear more consistent with those reported by Bove et al., who demonstrated reduced RV contractility in a swine model of chronic VO despite a considerably shorter follow-up period [23]. Further evidence of RV systolic impairment was provided by the significant reduction in TAPSE in both experimental groups, with the concomitant decrease in the TAPSE/RVSP ratio suggesting potential impairment of RV-PA coupling. Ventriculo-arterial coupling reflects the relationship between ventricular contractility and afterload and is commonly expressed as the ratio of end-systolic elastance to arterial elastance (Ees/Ea ratio). Although pressure–volume (P-V) loop analysis remains the gold standard for its assessment, several simplified surrogate markers have been proposed [24,25,26,27]. Among these, the TAPSE/sPAP ratio has gained increasing clinical relevance as a practical non-invasive parameter reflecting RV function and RV-PA coupling [28,29,30,31]. In our study, RVSP was used instead of sPAP, as the distal position of the Swan–Ganz catheter relative to the pulmonary artery band would have underestimated true RV afterload. Consistent with our findings, both clinical [27,32] and preclinical [33,34,35] studies have demonstrated RV-PA uncoupling in chronic PO, despite compensatory increases in contractility. Moreover, we observed a strong inverse correlation between the Fulton index and TAPSE/RVSP in this group, suggesting an association between greater RV hypertrophy and more severe RV-PA uncoupling. In contrast, the interpretation of RV-PA coupling in chronic VO remains challenging, with heterogeneous findings reported across studies. In a porcine model of neonatal PAB followed by TAP, Symakani et al. found preserved Ees associated with a reduction in Ea, resulting in higher RV-PA coupling. The authors attributed the reduction in afterload to pulmonary arterial unloading secondary to PR. Consequently, a reduction in Ea may result in pseudonormalization of RV-PA coupling despite possible underlying RV dysfunction [36]. The preserved contractility observed in their model, in contrast to the decline in TAPSE observed in our VO group, may be related to the preceding period of PO. As discussed by the authors, PO preceding or concomitant with VO may promote RV adaptation through increased contractility, thereby mitigating the functional consequences of VO [36]. In contrast, our VO model was not preceded by a period of PO, which may partly account for the differing results.

On the other hand, Latus et al. demonstrated impaired RV-PA coupling using invasive P-V loop analysis in patients with repaired tetralogy of Fallot (TOF) and chronic PR. Although both Ees and Ea increased during dobutamine stress, RV-PA coupling did not improve, with Ees remaining lower than Ea both at baseline and during inotropic stimulation, suggesting potential impairment of RV function [37].

Moreover, similar RV-PA coupling ratios may reflect substantially different underlying adaptive responses. Joosen et al. reported comparable Ees/Ea ratios between PO and VO despite marked differences in their individual components, with both Ees and Ea being higher in PO and lower in VO [5]. These findings emphasize the importance of considering the individual components of the coupling ratio, particularly when comparing different loading conditions. In our study, the TAPSE/RVSP ratio decreased in both groups. Although TAPSE showed a comparable decline, the increase in RVSP was substantially greater in PO than in VO, resulting in a lower ratio in the PO group. Increased RVSP in chronic VO has also been reported by Egbe et al. [38]. In adult patients with repaired TOF and PR, the authors found a reduced TAPSE/RVSP ratio, which was primarily driven by increased RVSP. The authors attributed this elevation to abnormal pulmonary arterial elastic properties [38]. Moreover, although VO primarily represents an increase in preload, progressive RV dilatation leads to increased wall stress and consequently contributes to increased RV afterload [3], which may also partly explain the increase in RVSP. In the study by Egbe et al. [38], however, TAPSE remained preserved, whereas in our VO group it decreased, suggesting impairment of systolic function. Nevertheless, it should be noted that TAPSE is both preload- and afterload-dependent. In chronic VO, TAPSE may remain preserved despite a progressive decline in Ees, potentially masking RV dysfunction. On the other hand, in patients with PH, TAPSE may be pseudonormalized by a reduction in afterload caused by concomitant tricuspid regurgitation [39]. Furthermore, TAPSE reflects only the longitudinal motion of the RV and is limited to the basal segment of the RV free wall [39]. This may be particularly relevant in patients with repaired TOF, who exhibit abnormal regional systolic function of the RV free wall involving the basal, mid, and apical segments [40], as well as RV outflow tract dysfunction related to postoperative scarring [41]. Therefore, TAPSE may not accurately reflect global RV systolic function.

Taken together, these findings suggest that despite having a potential role in risk stratification, RV-PA coupling should be interpreted with caution in chronic VO. This may be particularly relevant when using surrogate markers such as TAPSE/RVSP ratio.

### 4.3. Right Ventricular Diastolic Dysfunction

Besides its effects on systolic performance, chronic PO is also associated with RV diastolic dysfunction [3] as demonstrated in several experimental studies [42,43]. One year following induction of PO, animals demonstrated reduced transtricuspid E/A ratio consistent with impaired ventricular relaxation and altered early diastolic filling. This was accompanied by marked elevations in RAP and RVDP, suggesting progression toward more advanced RV diastolic dysfunction. Consistent with these findings, previous clinical studies in patients with PH have reported reduced transtricuspid E/A ratio [44] as well as a close correlation between elevated RAP and RV diastolic stiffness [45]. However, in our study, transtricuspid E/A ratio did not correlate with either RAP or RVDP, suggesting that changes in E/A ratio may not directly reflect RV filling pressures. The mechanisms underlying RV diastolic dysfunction in chronic PO are likely multifactorial, with both hypertrophy and fibrosis potentially contributing to this process [3]. However, while RV hypertrophy correlated with RV-PA coupling in our study, we found no significant correlation between the Fulton index and either RVDP or the transtricuspid E/A ratio. Similar findings have been reported in both preclinical and clinical studies. Borgdorff et al. showed that PAB-induced diastolic dysfunction, reflected by increased end-diastolic elastance (Eed), was independent of both RV hypertrophy and fibrosis [46]. Similarly, Joosen et al. reported increased Eed associated with elevated right ventricular end-diastolic pressure (RVEDP) but independent of myocardial hypertrophy [5]. These findings suggest that hypertrophic remodeling and fibrosis may not fully account for RV diastolic dysfunction in chronic PO. In this context, increased RV diastolic stiffness in patients with PH has been associated with intrinsic sarcomeric stiffening and reduced titin phosphorylation [47].

In contrast to PO, the reported effects of chronic VO on RV diastolic function appear more heterogeneous. Some investigators reported improved RV compliance and reduced stiffness three months following chronic VO, accompanied by normalization of RVEDP, suggesting successful structural adaptation to chronic chamber enlargement [23,48]. Conversely, Reddy et al. demonstrated persistently elevated RVEDP associated with early reductions in transtricuspid E/A ratio. Interestingly, E/A ratio subsequently normalized at six months follow-up, suggesting pseudonormalization rather than recovery of diastolic performance [49]. Our findings appear to occupy an intermediate position between these observations, as the reduction in transtricuspid E/A ratio was accompanied by a 60% increase in RAP, whereas RVDP remained unchanged. Restrictive RV physiology is common after TOF repair, with a reported prevalence ranging from 35% to 66%. It is characterized by reduced RV compliance, whereby the RV acts as a conduit between the right atrium and pulmonary artery during atrial contraction [50]. Hemodynamically, it has been characterized by increased RVEDP exceeding dPAP, resulting in forward flow into the pulmonary artery during late diastole. Accordingly, the presence of end-diastolic forward flow (EDFF) in the pulmonary artery has traditionally been considered the echocardiographic hallmark of restrictive RV physiology [50]. Given the conflicting evidence regarding its impact on long-term outcomes, two distinct phenotypes of restrictive RV physiology have been proposed [51]. The early-onset or “primary” phenotype develops in the immediate postoperative period and usually resolves within days to months after primary repair, although it may persist during mid-term follow-up [52]. In contrast, the late-onset or “secondary” phenotype develops later during follow-up and has been associated with an increased risk of adverse outcomes [52]. Despite the fact that EDFF has recently been shown not to be specific for restrictive RV physiology [50,52], neither EDFF nor simultaneous RV and pulmonary artery pressure measurements were assessed in our study, thereby limiting the interpretation of our findings. Moreover, both RAP and the E/A ratio are preload-dependent parameters. In patients with repaired TOF, the E/A ratio has been shown not to correlate with RVEDP [53], while increased filling pressures following TAP were not associated with increased Eed or RV stiffness in an experimental model of repaired TOF [36]. Therefore, although the increased RAP and decreased E/A ratio observed in our study suggest the presence of some degree of diastolic impairment, the available data are insufficient to establish restrictive RV physiology or to distinguish between a persistent early-onset and a late-onset phenotype.

### 4.4. Ventricular Interdependence and Left Ventricular Diastolic Dysfunction

Because both ventricles share the IVS and are enclosed within a relatively non-compliant pericardium, structural and functional changes affecting one ventricle inevitably influence the other, a phenomenon known as ventricular interdependence. Prolonged exposure to both pressure and volume overload can therefore induce secondary alterations in LV geometry and filling [3,54]. Elevation of RV pressures during chronic PO leads to prolonged RV contraction, a major determinant of interventricular dyssynchrony and leftward septal displacement. This subsequently impairs LV filling and reduces effective preload [3]. Previous experimental studies have shown reduced LV chamber volumes despite preserved intrinsic LV contractility [20], as well as increased LV stiffness [43]. Our findings were consistent with this concept, as animals exposed to chronic PO showed leftward septal shift as well as decreased LVIDd index, suggesting impaired LV filling secondary to altered ventricular geometry. Likewise, animals exposed to chronic VO demonstrated evidence of ventricular interdependence. However, LV involvement appeared more pronounced in this group, with a significantly lower LVIDd index and transmitral E/A ratio compared to the PO group. These findings suggest that chronic VO may compromise LV geometry and diastolic filling to a greater extent than chronic PO. Progressive RV dilation contributes not only through septal displacement, but also by increasing pericardial restraint and further limiting LV filling [55]. This may partly explain why secondary LV diastolic dysfunction has been consistently reported in experimental models of chronic pulmonary regurgitation and RV VO [21,55,56]. The combined effects of impaired LV filling and reduced RV forward flow can ultimately result in a low cardiac output state, explaining the reduction in MAP, LVSWI, LCWI and ScvO_2_ observed in both groups.

### 4.5. Right Ventricular Structural Remodelling

Chronic PO is associated with concentric hypertrophy, an initially adaptive mechanism aimed at reducing wall stress and preserving stroke volume in the setting of increased afterload. However, prolonged hypertrophy progressively becomes maladaptive, increasing myocardial oxygen demand and contributing to ventricular dysfunction [3]. Previous experimental studies have reported RV hypertrophy and fibrosis in PO models [19,43,57,58,59,60], with both structural alterations being associated with systolic and diastolic impairment [57,59,60]. In contrast, chronic VO induces a distinct remodeling pattern characterized predominantly by progressive chamber dilation and eccentric hypertrophy [3]. A recent meta-analysis reported that hypertrophy occurred early during chronic VO and appeared largely independent of both severity and duration, whereas fibrosis emerged later and has been consistently reported after approximately 90 days of exposure [61]. In accordance with these observations, both loading conditions resulted in myocardial hypertrophy, evidenced macroscopically and confirmed histologically. However, chronic PO induced a predominantly concentric remodeling pattern, characterized by increased IVSd thickness, RVW, and Fulton index, whereas chronic VO showed predominantly RV chamber dilation accompanied by a comparable increase in IVSd thickness, consistent with eccentric hypertrophy. Additionally, interstitial fibrosis was identified in both models. Beyond these structural changes, a particularly interesting finding of our study was the presence of large multinucleated cardiomyocyte-like cells, without inflammatory cells, which demonstrated distinct ventricular distribution patterns, being more frequent in the LV of the PO group and the RV of the VO group. Large multinucleated muscle cells have previously been described in skeletal muscle as part of the regenerative response following injury [62,63]. In contrast, adult mammalian cardiomyocytes are predominantly uni- or binucleated and are generally considered terminally differentiated with limited regenerative capacity [64]. Nevertheless, large multinucleated cells have been reported in a porcine model of chronic RV VO [65], and more recently, homotypic fusion of cardiomyocytes has been demonstrated in adult murine hearts subjected to LV PO, where it has been proposed as a potential contributor to hypertrophic remodeling [66,67]. To our knowledge, the preferential distribution of these cells in different ventricles according to the underlying pathophysiology represents a novel finding. While atrophic remodeling of the LV in chronic RV PO has been previously described [68], it remains unclear whether these multinucleated cells represent an adaptive response to mechanical stress or hypoxic injury, or whether they may constitute a precursor stage preceding fibrosis or myocardial atrophy. Their functional significance therefore remains uncertain, and whether their presence represents an early feature of maladaptive remodeling cannot be established from the present study. Future studies combining molecular characterization with longitudinal functional and hemodynamic assessment may determine whether their appearance is associated with specific stages of RV adaptation or maladaptation and whether corresponding non-histological changes can be identified.

### 4.6. Clinical Implications

In patients with CHD, chronic RV overload may remain clinically well tolerated for prolonged periods, making early recognition of maladaptive remodeling particularly challenging. Although these patients may be exposed to RV overload for considerably longer periods, the one-year follow-up allows direct comparison of RV adaptation to sustained PO and VO, which may help identify load-specific patterns of RV adaptation and contribute to a better understanding of the transition from adaptive to maladaptive remodeling [5]. Furthermore, the use of clinically established hemodynamic and echocardiographic parameters facilitates the translation of these findings to the clinical setting. A better understanding of RV adaptation to different loading conditions may facilitate the identification of more sensitive non-invasive markers of disease progression and contribute to improved surveillance and risk stratification. Ultimately, recognizing different patterns of RV maladaptation may support the development of load-specific therapeutic strategies [5], while earlier identification of maladaptive remodeling may help optimize the timing of reinterventions in patients with CHD.

### 4.7. Study Limitations

This study has several limitations that should be acknowledged. First, the relatively small sample size is an inherent limitation of large animal experimental studies and may have reduced statistical power for detecting subtle between-group differences. However, in accordance with animal welfare principles, we sought to minimize the number of animals used.

In addition, P-V loop analysis using conductance catheters was not performed. Although this technique is considered the gold standard in experimental settings, providing superior accuracy and comprehensive physiological information, its application in routine clinical practice remains limited. In this study, we aimed to use reliable, yet clinically translatable assessment methods currently used in routine clinical practice and readily applicable in point-of-care settings.

Moreover, although all assessments were performed under standardized conditions across animals, the fact that hemodynamic and blood gas measurements were obtained under general anesthesia, whereas transthoracic echocardiography was performed in awake animals, may influence the overall interpretation of the results.

The use of TAPSE/RVSP as a surrogate marker of RV-PA coupling also warrants consideration despite its clinical applicability and importance as a non-invasive parameter. As discussed above, TAPSE is influenced by loading conditions [39], while RVSP represents only a pressure-based estimate of RV afterload, unlike Ea, which incorporates stroke volume and reflects both the resistive and pulsatile components of RV afterload [69]. Therefore, changes in TAPSE/RVSP may not fully reflect the underlying changes in ventricular contractility and arterial load. Consequently, our findings regarding RV-PA coupling should be interpreted within the limitations of this surrogate marker.

Furthermore, the VO model included two different surgical approaches for creating pulmonary regurgitation. Although both procedures produced comparable regurgitation severity and similar acute hemodynamic profiles, subtle differences in long-term remodeling responses cannot be entirely excluded.

Another limitation relates to the histopathological analysis, as evaluation was performed only at the terminal one-year follow-up timepoint, preventing assessment of the temporal progression of myocardial remodelling. Moreover, myocardial hypertrophy and fibrosis were assessed descriptively without quantitative comparison between groups, limiting a more precise characterization of structural remodelling.

Finally, while the identification of large multinucleated cardiomyocyte-like cells with different ventricular distribution represents a potentially novel finding, the underlying mechanisms responsible for their formation remain unclear, as molecular characterization was beyond the scope of the current study. Further studies are required to determine whether these cells represent an adaptive regenerative response, or a maladaptive remodelling process associated with progressive myocardial injury.

## 5. Conclusions

One-year exposure to both pressure and volume overload resulted in distinct phenotypes of RV dysfunction. Both loading conditions led to impaired RV systolic function, with PO showing significant RV-PA uncoupling. Additionally, PO exhibited more pronounced RV diastolic dysfunction, whereas VO showed a greater impact on LV geometry and diastolic filling. Prolonged exposure to both loading conditions was associated with structural myocardial remodeling characterized by hypertrophy and fibrosis. In addition, the identification of multinucleated cardiomyocyte-like cells with preferential ventricular distribution according to overload physiology suggests the presence of a potentially novel remodeling mechanism that warrants further investigation. These findings highlight the importance of the underlying etiology of RV overload as a determinant of disease progression and may contribute to improved understanding of RV failure and future treatment optimization.

## Figures and Tables

**Table 1:** Demographic characteristics of animals, measured at baseline and one-year follow-up.

	PO (*n* = 6)			VO (*n* = 6)			PO vs. VO Follow-Up
Variables	Baseline Mean ± SD (Median)	Follow-Up Mean ± SD (Median)	*p*-Value	Baseline Mean ± SD (Median)	Follow-Up Mean ± SD (Median)	*p*-Value	
Weight (kg)	32.9 ± 11.7 (32.5)	51.2 ± 11.03 (51.0)	0.0313	32.7 ± 8.4 (36.5)	52.3 ± 8.2 (54.0)	0.0313	NS
Height (cm)	63.8 ± 13.9 (64.0)	85.8 ± 6.6 (86.0)	0.0313	63.7 ± 14.3 (70.0)	83.2 ± 4.4 (84.0)	0.0313	NS
BSA (m^2^)	0.65 ±0.19 (0.64)	0.97 ± 0.14 (0.96)	0.0313	0.64 ± 0.16 (0.71)	0.95 ± 0.1 (0.97)	0.0313	NS

BSA—body surface area; NS—not significant.

**Table 2:** Hemodynamic parameters measured through Swan–Ganz catheterization at baseline and one-year follow-up.

	PO (*n* = 6)		VO (*n* = 6)				PO vs. VO Follow-Up
Variables	Baseline Mean ± SD (Median)	Follow-up Mean ± SD (Median)	*p*-Value	Baseline Mean ± SD (Median)	Follow-Up Mean ± SD (Median)	*p*-Value	
SBP (mmHg)	107.2 ± 19.5 (105)	86.3 ± 13.2 (86.5)	NS	113.2 ± 15.8 (113.0)	99.8 ± 12.1 (101.5)	NS	NS
DBP (mmHg)	80 ± 15.3 (82.5)	45.0 ± 16.9 (43.5)	0.0625	80.8 ± 17.6 (78.0)	47.2 ± 8.0 (45.5)	0.0313	NS
MAP (mmHg)	89.2 ± 16.5 (90)	60.0 ± 14.6 (60.0)	0.0625	91.7 ± 16.8 (89.0)	66.2 ± 7.8 (63.5)	0.0313	NS
HR (beats/min)	108.2 ± 24.6 (103.5)	99.8 ± 23.6 (98.0)	NS	109 ± 16.6 (107.0)	103.0 ± 7.5 (102.5)	NS	NS
RAP (mmHg)	6.8 ± 1.6 (7)	13.3 ± 5.0 (14.0)	0.0313	6.5 ± 2.9 (7.5)	10.5 ± 2.4 (10.5)	0.0625	NS
RVSP (mmHg)	25.8 ± 5.6 (26.5)	51.7 ± 7.3 (49.0)	0.0313	26.5 ± 3.3 (28.0)	33.0 ± 3.3 (33.0)	0.0313	0.0022
RVDP (mmHg)	4.2 ±2.8 (4.5)	9.3 ± 1.0 (9.0)	0.0313	5.5 ± 2.4 (5.5)	4.7 ± 2.7 (5.5)	NS	0.0065
mRVP (mmHg)	11.5 ±3.2 (12)	23.0 ± 1.3 (22.5)	0.0313	12.5 ± 1.9 (12.5)	13.8 ± 1.7 (14.0)	NS	0.0022
sPAP (mmHg)	25 ± 4.6 (26.5)	28.8 ± 3.5 (28.0)	NS	26.0 ± 3.8 (27.0)	29.8 ± 3.7 (30.5)	NS	NS
dPAP (mmHg)	15.3 ± 4.0 (16)	15.7 ± 3.3 (15.5)	NS	17.5 ± 3.0 (18.0)	16.2 ± 4.4 (14.5)	NS	NS
mPAP (mmHg)	18.8 ± 4.1 (19.5)	19.7 ± 2.4 (20.0)	NS	20.7 ± 3.2 (21.0)	20.5 ± 3.9 (20.0)	NS	NS
PCWP (mmHg)	8.7 ± 1.6 (9)	10.0 ± 1.9 (10.0)	NS	9.3 ± 2.5 (9.5)	11.8 ± 1.6 (12.0)	NS	NS
SVI (mL/m^2^)	58.4 ± 14.5 (60)	30.7 ± 4.2 (29.1)	0.0313	58.6 ± 10.0 (56.4)	31.3 ± 14.7 (28.8)	0.0313	NS
CI (L/min/m^2^)	6.2 ±1.8 (6.2)	3.0 ± 0.6 (3.0)	0.0313	6.4 ± 1.6 (6.4)	3.2 ± 1.5 (3.0)	0.0313	NS
SVRI (dyn.s.cm^−5^.m^2^)	1133.8 ± 328.5 (1164.5)	1341.3 ± 668.8 (1238.5)	NS	1082.2 ± 140.2 (1043.5)	1651.3 ± 1102.1 (1379.5)	NS	NS
PVRI (dyn.s.cm^−5^.m^2^)	131.2 ± 25.3 (130.0)	244.0 ± 103.7 (229.0)	NS	158.2 ± 105.3 (141.5)	235.3 ± 182.1 (170.0)	NS	NS
LCWI (kg.m/m^2^)	6.8 ± 2.1 (7.1)	1.9 ± 0.2 (2.0)	0.0313	7.5 ± 3.2 (6.8)	2.4 ± 1.5 (2.0)	0.0313	NS
LVSWI (g.m/m^2^)	64.1 ± 20.3 (63.2)	20.9 ± 7.1 (20.4)	0.0313	66.7 ± 21.6 (66.0)	23.8 ± 14.7 (19.5)	0.0313	NS
RCWI (kg.m/m^2^)	1.1 ± 0.5 (1.0)	0.26 ± 0.1 (0.3)	0.0313	1.2 ± 0.5 (1.1)	0.5 ± 0.3 (0.3)	0.0313	NS
RVSWI (g.m/m^2^)	9.8 ± 4.2 (9.8)	2.6 ± 1.4 (2.9)	0.0313	10.9 ± 3.3 (11.0)	4.4 ± 3.3 (3.0)	0.0313	NS

PO—pressure overload; VO—volume overload; SBP—systolic blood pressure; DBP—diastolic blood pressure; MAP—mean arterial pressure; HR—heart rate; RAP—right atrial pressure; RVSP—right ventricular systolic pressure; RVDP—right ventricular diastolic pressure; mRVP—mean right ventricular pressure; sPAP—systolic pulmonary artery pressure; dPAP—diastolic pulmonary artery pressure; mPAP—mean pulmonary artery pressure; PCWP—pulmonary capillary wedge pressure; SVI—stroke volume index; CI—cardiac index; SVRI—systemic vascular resistance index; PVRI—pulmonary vascular resistance index; LCWI—left cardiac work index; LVSWI—left ventricular stroke work index; RCWI—right cardiac work index; RVSWI—right ventricular stroke work index; NS—not significant.

**Table 3:** Parameters measured through blood gas analyses at baseline and one-year follow-up.

	PO (*n* = 6)			VO (*n* = 6)			PO vs. VO Follow-Up
Variables	Baseline Mean ± SD (Median)	Follow-Up Mean ± SD (Median)	*p* Value	Baseline Mean ± SD (Median)	Follow-Up Mean ± SD (Median)	*p* Value	
ScvO_2_ (%)	92.7 ± 2.6 (92.5)	79.5 ± 9.5 (80.5)	0.0313	86.2 ± 5.4 (83.5)	80.7 ± 5.0 (80.5)	0.0313	NS
pH	7.45 ± 0.13 (7.43)	7.40 ± 0.07 (7.39)	NS	7.39 ± 0.05 (7.39)	7.39 ± 0.05 (7.4)	NS	NS
pO_2_ (mmHg)	198.7 ± 21.6 (202.0)	94.2 ± 28.7 (101.5)	0.0313	168.5 ± 35.7 (174.5)	117.0 ± 26.4 (112.5)	0.0313	NS
pCO_2_ (mmHg)	44.7 ± 13.6 (46.0)	46.0 ± 5.8 (47.0)	NS	47.9 ± 4.7 (48.0)	47.1 ± 2.9 (47.1)	NS	NS
SaO_2_ (%)	99.8 ± 0.4 (100.0)	95.2 ± 4.8 (96.5)	0.0625	99.3 ± 0.8 (99.5)	97.8 ± 0.8 (98.0)	NS	NS
HCO_3_^−^ (mmol/L)	29.3 ± 3.9 (29.0)	28.2 ± 1.3 (28.5)	NS	29.4 ± 2.7 (29.3)	28.7 ± 1.3 (28.8)	NS	NS
BE (mmol/L)	5.2 ± 3.3 (4.5)	3.7 ± 1.9 (4.0)	NS	4.5 ± 3.5 (4.5)	3.7 ± 2.3 (3.5)	NS	NS
Lactate (mmol/L)	0.6 ± 0.3 (0.6)	0.8 ± 0.4 (0.8)	NS	0.4 ± 0.2 (0.4)	0.8 ± 0.9 (0.5)	NS	NS
Hb (g/dL)	6.7 ± 1.2 (6.8)	6.7 ± 0.8 (6.9)	NS	7.0 ± 1.1 (7.2)	6.9 ± 0.7 (6.8)	NS	NS
Hct (%)	19.8 ± 3.6 (20.0)	19.8 ± 2.3 (20.5)	NS	20.7 ± 3.1 (21.0)	20.2 ± 2.0 (20.0)	NS	NS

ScvO_2_—central venous oxygen saturation; pO_2_—oxygen partial pressure; pCO_2_—carbon dioxide partial pressure; SaO_2_—arterial oxygen saturation; HCO_3_^−^—bicarbonate; BE—base excess; Hb—hemoglobin; Hct—hematocrit; NS—not significant.

**Table 4:** Transthoracic echocardiographic parameters at baseline and one-year follow-up.

	PO (*n* = 6)			VO (*n* = 6)			PO vs. VO Follow-Up
Variables	Baseline Mean ± SD (Median)	Follow-Up Mean ± SD (Median)	*p* Value	Baseline Mean ± SD (Median)	Follow-Up Mean ± SD (Median)	*p* Value	
MAPSE (mm)	14 ± 0.6 (14.0)	12.0 ± 2.1 (11.5)	NS	16.5 ± 3.7 (16.0)	12.8 ± 2.4 (12.9)	0.0625	NS
TAPSE (mm)	16.3 ± 1.1 (17.0)	8.8 ±1.2 (8.9)	0.0313	14.9 ± 3.2 (16.1)	9.8 ± 1.3 (9.8)	0.0313	NS
Transmitral E/A ratio	1.27 ± 0.18 (1.2)	1.22 ± 0.19 (1.2)	NS	1.35 ± 0.26 (1.35)	0.98 ± 0.10 (0.95)	0.0313	0.0152
Transtricuspid E/A ratio	1.13 ± 0.15 (1.2)	0.88 ± 0.11 (0.88)	0.0313	1.47 ± 0.27 (1.44)	0.76 ± 0.12 (0.75)	0.0313	0.0931
LVEF (%)	65.8 ± 7.5 (62.5)	60.5 ± 6.2 (60.0)	NS	55.4 ± 4.9 (55.6)	57.4 ± 8.1 (59.1)	NS	NS
LVFS (%)	36.3 ± 5.8 (34.0)	31.5 ± 4.3 (30.5)	NS	28.3 ± 2.6 (28.0)	31.1 ± 3.3 (30.1)	NS	NS
LVIDd index (cm/m^2^)	5.9 ± 0.7 (6.1)	3.8 ± 0.6 (3.8)	0.0313	5.4 ± 0.6 (5.3)	2.7 ± 0.9 (2.7)	0.0313	0.0411
RVIDd index (cm/m^2^)	n.d.	2.2 ± 0.7 (2.3)	-	n.d.	2.7 ± 0.7 (2.6)	-	NS
IVSd thickness (cm)	0.71 ± 0.14 (0.76)	1.14 ± 0.14 (1.12)	0.0313	0.86 ± 0.17 (0.9)	1.14 ± 0.06 (1.14)	0.0313	NS
TAPSE/RVSP ratio (mm/mmHg)	0.66 ± 0.13 (0.61)	0.17 ± 0.02 (0.17)	0.0313	0.57 ± 0.12 (0.59)	0.30 ± 0.06 (0.29)	0.0313	0.0022
Leftward septal shift (no. of animals)	0	6	-	0	6	-	-

MAPSE—mitral annular plane systolic excursion; TAPSE—tricuspid annular plane systolic excursion; LVEF—left ventricular ejection fraction; LVSF—left ventricular fractional shortening; LVIDd—left ventricular internal diameter at end-diastole; RVIDd—right ventricular internal diameter at end-diastole; IVSd—interventricular septum at end-diastole; RVSP—right ventricular systolic pressure; n.d.—not determined; NS—not significant.

**Table 5:** Macroscopic characteristics of the hearts at one-year follow-up.

	PO (*n* = 6)	VO (*n* = 6)	PO vs. VO Follow-Up
Variables	Follow-Up Mean ± SD (Median)	Follow-Up Mean ± SD (Median)	
BSA (m^2^)	0.972 ± 0.14 (0.96)	0.952 ± 0.1 (0.97)	NS
HW (g)	234.2 ± 50.3 (226.0)	196.0 ± 14.7 (198.0)	0.0649
HW/BSA (g/m^2^)	240.7 ± 31.3 (243.6)	208.7 ± 35.6 (201.4)	NS
RVW (g)	93.2 ± 24.4 (87.5)	65.2 ± 13.0 (63.0)	0.0260
RVW/BSA (g/m^2^)	95.6 ± 16.7 (94.0)	70.1 ± 21.0 (63.3)	0.0649
RVW/HW	0.40 ± 0.02 (0.40)	0.33 ±0.04 (0.33)	0.0200
LV + IVS (g)	136.0 ± 32.4 (134.0)	130.8 ± 6.0 (130.5)	NS
LV + IVS/BSA (g/m^2^)	138.8 ± 16.7 (138.1)	138.6 ± 15.6 (138.0)	NS
LV + IVS/HW	0.58 ± 0.05 (0.59)	0.67 ± 0.04 (0.68)	0.0087
RV/LV + IVS (Fulton index)	0.69 ± 0.08 (0.70)	0.50 ± 0.10 (0.48)	0.0087

BSA—body surface area; HW—heart weight; RVW—right ventricular weight; LV + IVS—combined left ventricular plus interventricular septum weight; NS—not significant.

## Data Availability

The datasets used and analyzed during the current study are available from the corresponding author upon reasonable request.

## Abbreviations

The following abbreviations are used in this manuscript:

RV	Right ventricle
LV	Left ventricle
PH	Pulmonary hypertension
CHD	Congenital heart disease
LVF	Left ventricular failure
RVF	Right ventricular failure
PO	Pressure overload
VO	Volume overload
PAB	Pulmonary artery banding
PA	Pulmonary artery
PR	Pulmonary regurgitation
TAP	Transannular patch
RVOT	Right ventricular outflow tract
SBP	Systolic blood pressure
DBP	Diastolic blood pressure
MAP	Mean arterial pressure
RVSP	Right ventricular systolic pressure
RVDP	Right ventricular diastolic pressure
mRVP	Mean right ventricular pressure
sPAP	Systolic pulmonary artery pressure
dPAP	Diastolic pulmonary artery pressure
mPAP	Mean pulmonary artery pressure
RAP	Right atrial pressure
PCWP	Pulmonary capillary wedge pressure
CI	Cardiac index
SVI	Stroke volume index
SVRI	Systemic vascular resistance index
PVRI	Pulmonary vascular resistance index
RVSWI	Right ventricular stroke work index
LVSWI	Left ventricular stroke work index
RCWI	Right cardiac work index
LCWI	Left cardiac work index
pO_2_	Oxygen partial pressure
pCO_2_	Carbon dioxide partial pressure
SaO_2_	Arterial oxygen saturation
HCO_3_^−^	Bicarbonate
BE	Base excess
Hb	Hemoglobin
Hct	Hematocrit
ScvO_2_	Central venous oxygen saturation
TTE	Transthoracic echocardiography
TAPSE	Tricuspid annular plane systolic excursion
MAPSE	Mitral annular plane systolic excursion
E/A ratio	Early-to-late flow velocity ratio
LVIDd	Left ventricular internal diameter at end-diastole
RVIDd	Right ventricular internal diameter at end-diastole
IVSd	Interventricular septum at end-diastole
PLAX	Parasternal long-axis
BSA	Body surface area
LVEF	Left ventricular ejection fraction
LVFS	Left ventricular fractional shortening
RV-PA	Right ventricular-pulmonary arterial
HW	Heart weight
RVW	Right ventricular weight
LV + IVS	Left ventricular + interventricular septum weight
IVS	Interventricular septum
PSAX	Parasternal short-axis
ECG	Electrocardiogram
Ees	End-systolic elastance
Ea	Arterial elastance
P-V	pressure–volume
TOF	Tetralogy of Fallot
Eed	End-diastolic elastance
PVEDP	Right ventricular end-diastolic pressure
EDFF	End-diastolic forward flow
